# Correction: Rapidly evolving protointrons in *Saccharomyces* genomes revealed by a hungry spliceosome

**DOI:** 10.1371/journal.pgen.1008854

**Published:** 2020-05-27

**Authors:** Jason Talkish, Haller Igel, Rhonda J. Perriman, Lily Shiue, Sol Katzman, Elizabeth M. Munding, Robert Shelansky, John Paul Donohue, Manuel Ares Jr.

In the Data Availability statement and the Mapping and analysis of RNAseq data subsection of Materials and Methods, the accession number for RNAseq data deposited in GEO is listed incorrectly. The correct accession number is: GSE90105.
