# Supplementary figures and images for: Rapidly evolving protointrons in Saccharomyces genomes revealed by a hungry spliceosome
Source: PLoS Genet. 2019 Aug 22;15(8):e1008249. doi: 10.1371/journal.pgen.1008249 (PMC6726248; doi:10.1371/journal.pgen.1008249)

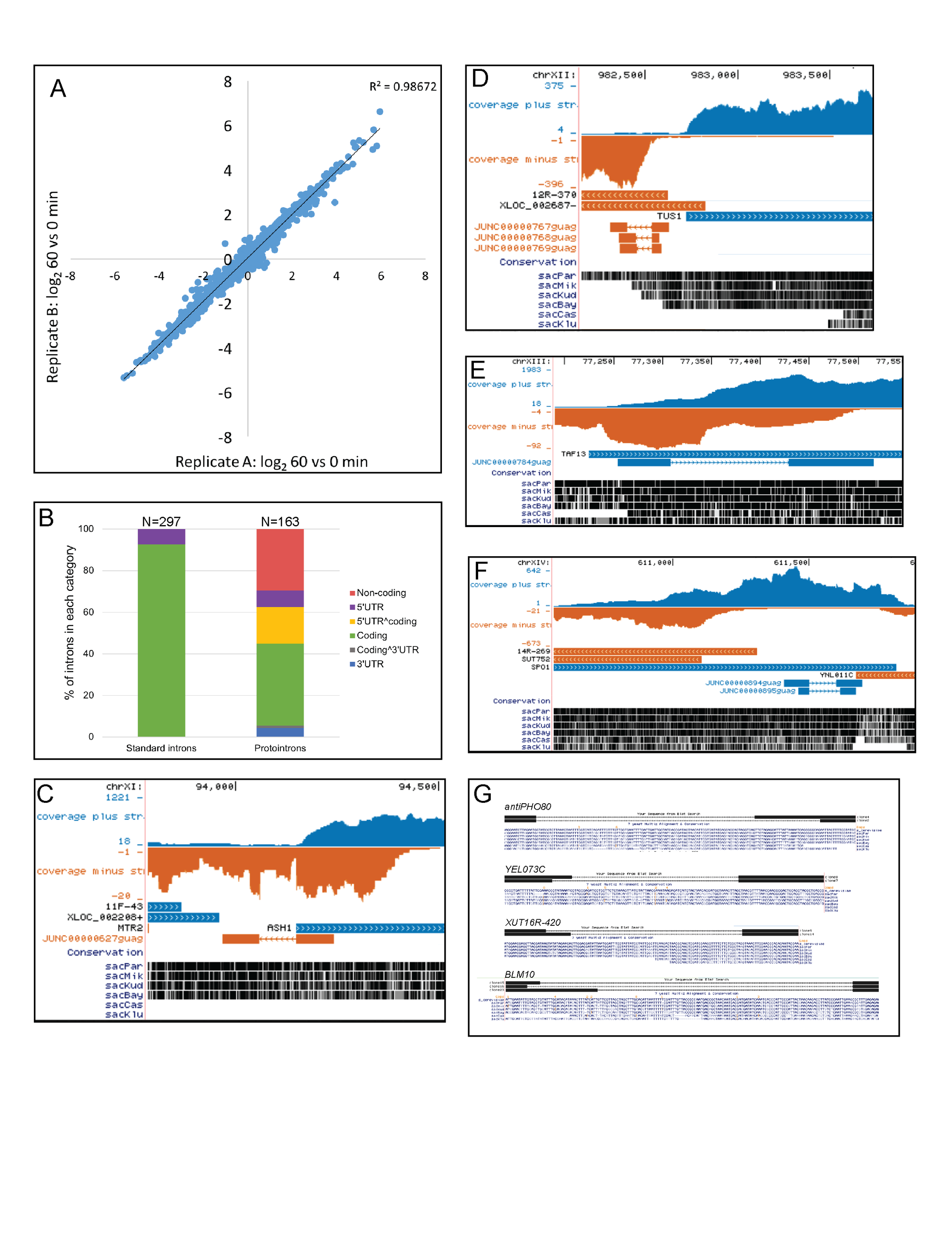

Supplement: S1 Fig — (A) Coherence of gene expression changes after 60 minute rapamycin treatment between the two replicate experiments. Log2ratio of treatment to control read coverage over genes was plotted giving an R2 value of ~0.99. Supplemental to Fig 1A. (B) Percentage of standard introns and protointrons that are located in non-coding, 5’UTR, 5’UTR^coding, coding, coding^3’UTR and 3’UTR regions. (C) Coverage tracks showing transcription through the genomic locus upstream of ASH1 where the antiASH1 protointron is located. Supplemental to Fig 1B and 1C. (D) Coverage tracks showing transcription through the genomic locus upstream of TUS1 where the XUT12R-370 protointron is located. Supplemental to Fig 1B and 1C. (E) Coverage tracks showing transcription through the genomic locus of TAF13 where the TAF13 protointron is located. Supplemental to Fig 1B and 1C. (F) Coverage tracks showing transcription through the genomic locus of SPO1 where the ncSPO1 protointron is located. Supplemental to Fig 1B and 1C. (G) Alignment of sequenced RT-PCR products showing the location of protointrons with unusual 5’ ss. Supplemental to Fig 1D. (TIF) [file pgen.1008249.s001.tif]
